# Clinical features and prognosis of cardiac metastatic tumors

**DOI:** 10.1186/s12885-023-11733-1

**Published:** 2023-12-15

**Authors:** Zhongqiao Lin, Huazhen Xiao, Jun Liu, Ling Chen, Huishan Zhang, Yufang Huang, Yu Chen, Jing Lin

**Affiliations:** 1 Phase I Clinical Trial Ward, Clinical Oncology School of Fujian Medical University, Fujian Cancer Hospital, Fuzhou, Fujian Province 350000 China; 2 Department of Medical Oncology, Clinical Oncology School of Fujian Medical University, Fujian Cancer Hospital, Fuzhou, Fujian Province 350000 China; 3 Cancer Bio-Immunotherapy Center, Clinical Oncology School of Fujian Medical University, Fujian Cancer Hospital, Fuzhou, Fujian Province 350000 China; 4 Department of Cardiology, Shengli Clinical Medical College of Fujian Medical University, Fujian Provincial Hospital, Fuzhou, Fujian Province 350000 China

**Keywords:** Tumor, Cardiac metastasis, Cancer survivorship, Cardiac function, Echocardiography, Efficacy evaluation

## Abstract

**Background:**

This study aimed to explore the clinical features and prognosis of cardiac metastatic tumors. In addition, whether continuing antitumor therapy after the development of cardiac metastases can benefit patients and the response of cardiac metastases were investigated.

**Methods:**

A retrospective analysis was conducted on patients with malignancies who were admitted to Fujian Cancer Hospital and Fujian Provincial Hospital from January 2007 to September 2022, and the follow-up period ended in March 2023. Clinical data were gathered, treatment efficacy was evaluated, and survival analysis was performed.

**Results:**

After the patients developed cardiac metastasis, the overall 30-day, 3-month, 6-month, and 12-month survival rates were 85.00%, 59.00%, 51.00% and 38.00%, respectively. With continued treatment, the average survival time was 27.33 months (95% confidence interval [CI]: 16.88–37.79), which exceeded the 6.6 months (95% confidence interval [CI]: 0.03–13.69) observed for patients who withdrew from treatment (*P* < 0.001). The responses of cardiac metastases corresponded to the responses of the primary tumors. Patients with a cardiac response had a median survival time of 55.60 months, which exceeded the 13.40 months observed for those without a cardiac response. However, there was no significant difference (*P* = 0.375).

**Conclusions:**

In conclusion, continuing antitumor therapy after the development of cardiac metastases can significantly prolong patient survival. Cardiac metastases and primary tumors respond consistently to antitumor treatment. The risk of death due to heart failure in cancer patients with cardiac metastases needs to be further investigated.

## Background

Cardiac tumors, whether benign or malignant, are relatively rare among oncological diseases, with an overall prevalence of no more than 0.33% [1]. Cardiac tumors can also be classified as primary or metastatic, depending on their origin. The frequency of primary cardiac tumors is approximately 1.38/100000, and a recent study has shown that 90% of primary cardiac tumors are benign, with most being myxomas [2]. Metastatic cardiac tumors are described as being approximately 22 to 132 times more common than primary cardiac tumors [3–5]. However, the incidence of cardiac metastases reported in the literature is diverse, ranging from 2.3% to 18.3% (average incidence of 7.1%) among autopsies of cancer patients [6, 7]. The symptoms of cardiac tumors are nonspecific and can mimic the manifestations of many other heart diseases, making them difficult to diagnose and cure [8]. Twelve percent of oncology patients with uncomfortableness in the heart were found to have tumor metastases in the heart or pericardium at autopsy [9]. The occurrence of cardiac metastases often means that the tumor has progressed to a terminal stage, and whether continued treatment benefits the patient is worth studying [10]. When treatment is continued, whether a valid cardiac response associated with an increase in patient survival also needs to be indicated. Currently, most clinical studies of cardiac metastatic tumors have been case reports [11–15]. The overviews of the clinical features as well as the prognosis of patients with cardiac metastatic tumors are limited. This study reviewed the clinical data of cancer patients diagnosed in Fujian Cancer Hospital and Fujian Provincial Hospital from 2007 to 2022, exploring the clinical features and prognosis of cardiac metastatic tumors.

## Materials and methods

A retrospective analysis was conducted on patients with malignancies who were admitted to Fujian Cancer Hospital and Fujian Provincial Hospital from January 2007 to September 2022, and the follow-up period ended in March 2023. Patients with malignancies were diagnosed through detailed medical history, complete physical examination and pathologic results. Cardiac metastases were confirmed by imaging examinations (echo, cardiac CT, cardiac MRI, etc.), and all tumors had metastasized to the heart and/or invaded the pericardium from external sources. Both patients who continued treatment and those who withdrew from treatment were included. This study obtained only clinical information (medical history, clinical examinations and prognosis) and was carried out in accordance with the standards of the Declaration of Helsinki. Informed consent was obtained from all patients or their legal guardians. This study was approved by the Ethics Committee of Fujian Cancer Hospital and the Ethics Committee of Fujian Provincial Hospital.

Efficacy evaluation of antitumor treatments for primary tumors and metastases was performed. For solid tumors, it was based on Response Evaluation Criteria In Solid Tumors (RECIST version 1.1) [16]; for lymphohematopoietic tumors, it was based on Lugano 2014 standard [17]. Efficacy grades were divided as follows: complete response (CR), partial response (PR), stable disease (SD) and progressive disease (PD). The cardiac response is defined as an evaluation of the efficacy of the cardiac metastases as PR or CR after treatment. In echocardiography data, the left ventricular ejection fraction (LVEF) reflects cardiac systolic function, and the early diastolic transmitral flow velocity/early diastolic mitral annular velocity (E/e’) ratio reflects cardiac diastolic function.

All statistical analyses were performed using SPSS v27.0. Continuous variables are presented as the mean ± SD when normally distributed and as the median (interquartile range) otherwise. Descriptive parameters such as frequencies and percentages were calculated for categorical data. For normally distributed continuous variables, between-group differences were performed with independent-sample t tests; for nonnormally distributed data, Wilcoxon rank-sum tests were used. The 1-, 3-, 6-, and 12-month survival outcomes were examined, and comparisons were evaluated by Kaplan‒Meier curves and log-rank statistics. The survival of the following groups was compared: continued treatment versus discontinued treatment and cardiac response versus no cardiac response. All survival data were right-censored. All tests were 2-sided, and an alpha value of 0.05 was used to define statistical significance.

## Results

### Clinical features of patients with cardiac metastases

A total of 41 patients with metastatic cardiac malignancies were included in the current study. As shown in Tables 1 and 2, the median age of the 41 patients was 60 years (interquartile range: 49.50 to 66.50 years), and there were 16 women and 25 men. The median time of development of cardiac metastasis was 4.30 months [interquartile range: 0.15–18.15 months]. Twelve of the 41 patients had cardiac metastasis when primary tumors were diagnosed. Twenty-seven patients continued to receive treatment, and the other 14 patients withdrew from treatment. Of those being treated, 13 patients chose chemotherapy alone; 11 patients chose chemotherapy combined with immunotherapy, radiation or targeting drugs; 2 patients chose surgery combined with radiation or targeting drugs; 1 patient chose targeting drugs alone; and 1 patient chose radiation alone.

**Table 1:** Clinical features of patients with metastatic cardiac tumors

Item	No. of patients or cases (n)
**Sex**	
Male	25
Female	16
**Age, years**	
< 60	19
≥ 60	22
Average	56.20 (interquartile range: 49.50 – 66.50)
Median	60.00
**Type of primary malignancy**	
Lymphoma	10
Lung cancer	7
Cervical cancer	3
Renal cancer	3
Melanoma	2
Esophageal cancer	4
Colorectal cancer	2
Soft tissue sarcoma	2
Liver cancer	2
Bone sarcoma	2
Uncertain pathologic type	2
**Site of cardiac metastasis**	
Left atrium	4 cases
Left ventricle	14 cases
Right atrium	5 cases
Right ventricle	16 cases
Interventricular septum	4 cases
Pericardium	13 cases
Other	5 cases
**Cardiac symptoms**	
Yes	12
No	29
**Pericardial effusion**	
Yes	15
No	26
**Hypertension**	
Yes	10
No	31
**Diabetes**	
Yes	5
No	36
**History of smoking**	
Yes	8
No	33
**History of alcohol**	
Yes	4
No	37
**History of heart disease**	
Yes	7
No	34
**Abnormal ECG after development of cardiac metastasis**	
Yes	17
No	24
**Valvular regurgitation after development of cardiac metastasis**	
Yes	25
No	2
Undetected	14

**Table 2:** Clinical features of cardiac metastases

Item	Average	Median
Time of development to cardiac metastasis, months	15.27 (interquartile range: 0.15–18.15)	4.30
Long diameter of cardiac metastasis, cm	3.84 ± 2.00	3.90
Cross-sectional area of cardiac metastasis, cm^2^	12.86 ± 11.36	9.40
Overall survival from cardiac metastasis to death, months	19.70 (95% CI: 12.14–27.27)	7.70
Survival of patients continuing treatments, months	27.33 months (95% CI: 16.88–37.79)	13.60
Survival of patients withdraw treatments, months	6.60 (95% CI: 0.03–13.69)	2.10

A large plurality of primary malignancies was lymphoma (10 of 41), followed by lung cancer (7 of 41), esophageal cancer (4 of 41), cervical cancer (3 of 41), renal cancer (3 of 41), melanoma (2 of 41), colorectal cancer (2 of 41), osteosarcoma (2 of 41), soft tissue tumors (2 of 41), and other unclassified primary tumors (2 of 41). The average length of the metastatic cardiac tumors was 3.84 ± 2.00 cm, and the average cross-sectional area was 12.86 ± 11.36 cm^2^. Most cardiac metastases were single lesions, while another 3 patients had more than 3 cardiac metastases. The most common location of the cardiac metastases was the right ventricle (16 cases), followed by the left ventricle (14 cases), pericardium (13 cases), right atrium (5 cases), left atrium (4 cases), and interventricular septum (4 cases); in the other 5 cases, the locations were difficult to distinguish. Ten patients had both ventricular and pericardial involvement.

Seven patients had a history of heart disease. Ten patients had hypertension, and 5 patients had diabetes. A small number of patients had a history of smoking or drinking. A history of cardiovascular disease and cardiovascular risk factors did not seem to be associated with the development of cardiac metastases. After cardiac metastases developed, 17 patients had electrocardiographic abnormalities, 15 patients had hydropericardium, and only 12 patients had cardiac symptoms such as chest distress and tachypnea. Echocardiography showed that 25 patients had more than one instance of valvular regression. However, no decrease in ejection fraction was observed. The details are shown in Tables 1 and 2.

### Echocardiographic features of 8 patients

Eight patients had available serial echocardiography reports. As shown in Table 3, all 8 patients had variable increases in atrial and ventricular volumes compared with those before the development of cardiac metastases, but these differences were not marked. All 8 patients had valvular regurgitation after cardiac metastasis. There was a tendency for myocardial thickening, but the difference was not significant (*P* > 0.05). There was also no significant change in LVEF (*P* > 0.05) among the 8 patients, but three patients displayed E/e’ > 12 after cardiac metastasis, which suggested the potential possibility of diastolic insufficiency.

**Table 3:** Cardiac ultrasound results before and after cardiac metastasis developed in 8 patients

ID	Status	Cardiac symptoms	Pericardial effusion		Before (mm)									After(mm)					
				HR	LAd	LVd	IVSd	PLVWd	LVEF	E/e’	VR	HR	LAd	LVd	IVSd	PLVWd	LVEF	E/e’	VR
1	Died	Yes	No	88	25.0	42.0	11.7	9.5	62	-	-	89	35.0	43.0	11.7	10.7	56	-	+
3	Alive	No	No	63	31.1	35.5	11.6	11.6	60	12.6	-	68	30.0	35.7	10.3	10.5	61	14.1	+
5	Died	Yes	No	87	27.8	42.0	8.4	8.0	63	10.0	+	82	29.2	33.0	9.8	9.5	59	9.4	+
7	Died	No	No	54	32.0	40.0	12.5	14.2	60	-	-	81	33.0	40.0	12.3	14.0	59	-	+
9	Died	Yes	Yes	95	32.7	44.2	10.3	10.1	56	9.1	+	97	32.0	43.3	11.4	10.1	62	12.0	+
26	Died	Yes	Yes	44	36.7	39.9	13.1	10.9	58	10.6	+	79	35.7	40	22.7	14.4	56	12.8	+
34	Died	Yes	Yes	84	32.7	46.9	13.6	11.5	61	8.0	+	127	27.3	37.9	10.3	8.8	59	8.1	+
41	Died	No	No	88	24.0	37.0	13.0	10.7	63	-	+	76	41.2	45.7	12.2	12.3	59	-	+

*HR* heart rate, *Lad* left atrial diameter, *LVd* left ventricular diameter, *IVSd* interventricular septum diameter, *PLVWd* left ventricular posterior wall diameter, *CO* cardiac output, *LVEF* left ventricular ejection fraction, *E/e’* early diastolic transmitral flow velocity/early diastolic mitral annular velocity ratio, *VR* valvular regurgitation

### Survival analysis of patients with cardiac metastases

**Fig. 1:**
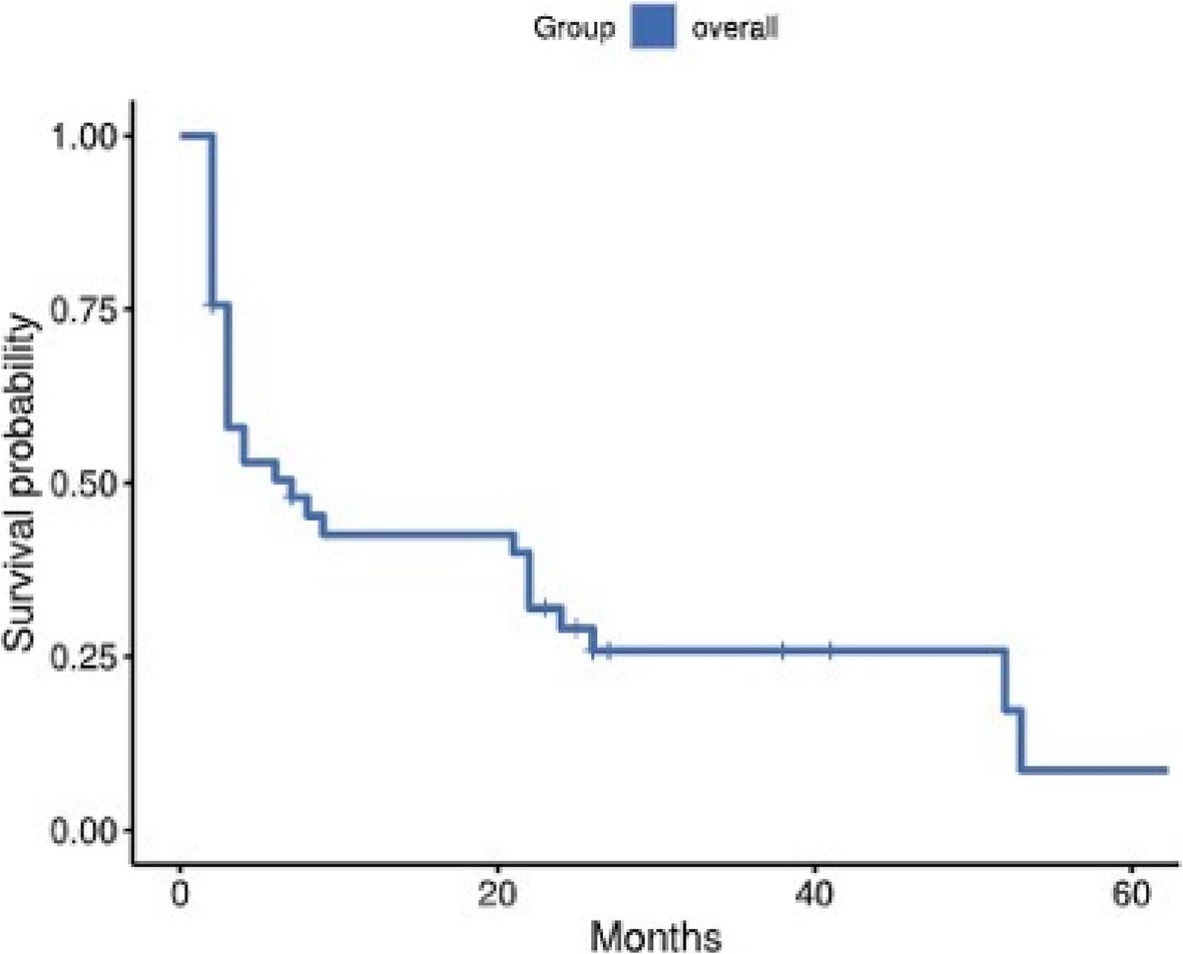
Overall survival after the development of cardiac metastases

**Fig. 2:**
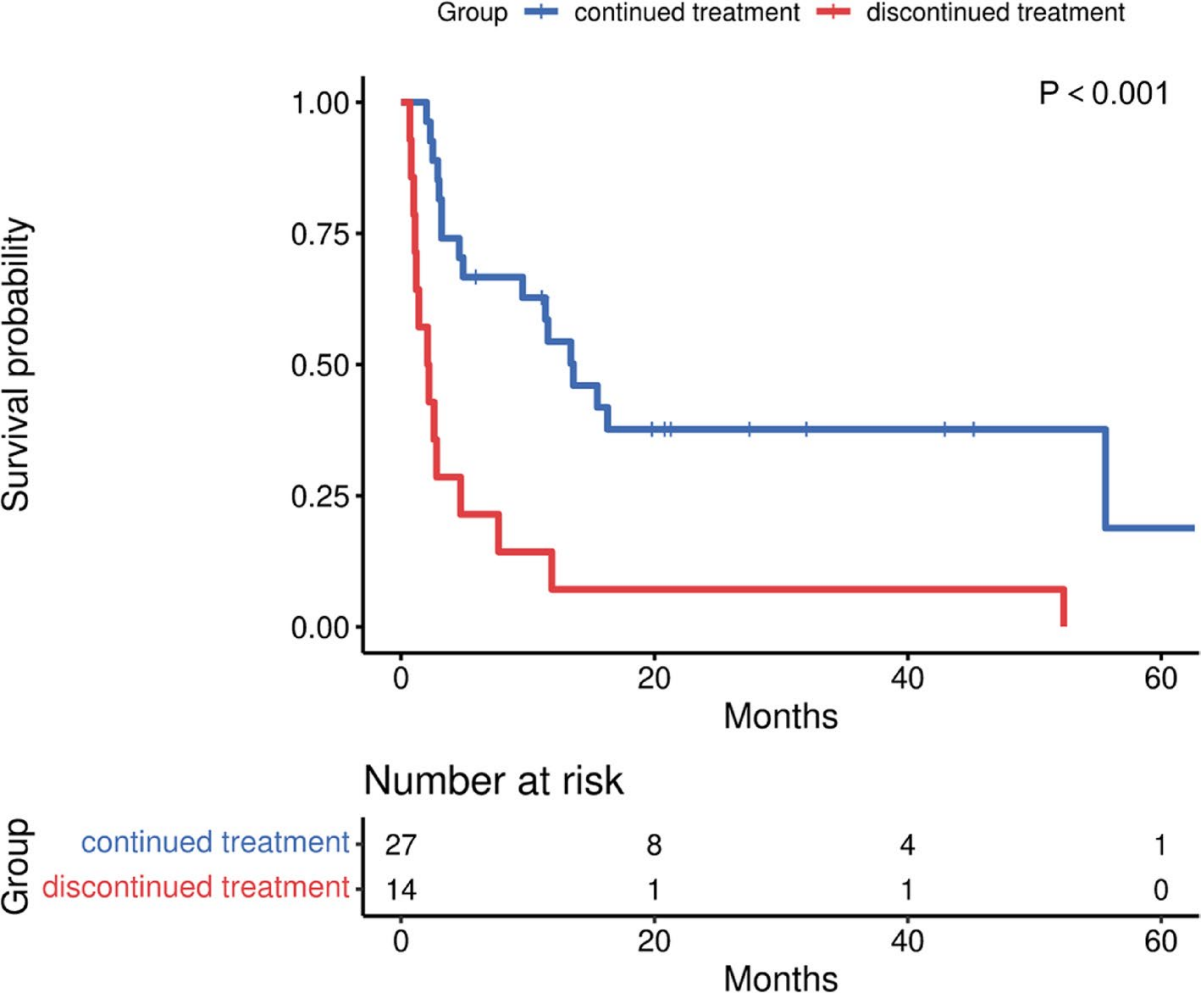
Effect of continuation or discontinuation of treatment on survival after cardiac metastasis development

**Fig. 3:**
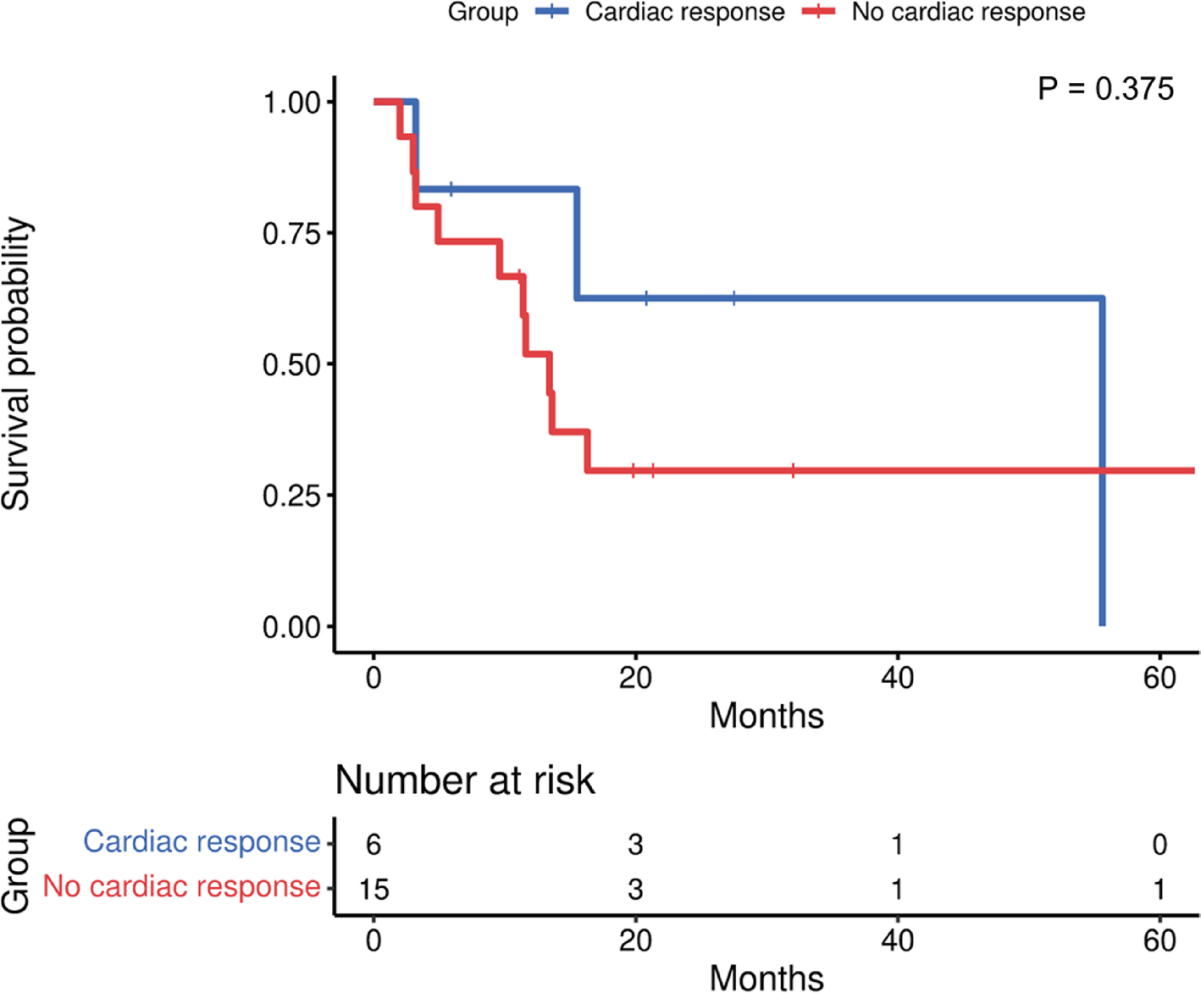
Effect of cardiac response on survival after cardiac metastasis development

As of 2023.03.01, 9 patients were still alive. After cardiac metastases, the overall 1-, 3-, 6-, and 12-month survival rates were 85.00%, 59.00%, 51.00% and 38.00%, respectively (Fig. 1). The overall mean survival time of the patients was 19.70 months (95% confidence interval [CI]: 12.14–27.27), and the median time was 7.48 months. With continued treatment, the average survival time was 27.33 months (95% confidence interval [CI]: 16.88–37.79), and the median time was 13.60 months. Without continued treatment, the average survival time was 6.60 months (95% confidence interval [CI]: 0.03–13.69), and the median time was 2.10 months. There was a significant survival difference (*P* < 0.001) between patients with or without continued treatment, and patients who continued treatment had a longer survival time (Fig. 2). Among patients who continued treatment, those with a cardiac response had a median survival time of 55.60 months, which was longer than the 13.40 months observed in those without a cardiac response. However, there was no survival difference (*P* = 0.375) based on the treatment response of cardiac metastasis (Fig. 3).

### Efficacy evaluations of patients with cardiac metastases

Although 27 patients continued treatment, only 23 patients underwent efficacy evaluations for their primary tumors, and 19 patients had efficacy evaluations for cardiac metastases and other metastases (Table 4). The efficacy evaluations for the primary tumors revealed that 4 patients achieved PD, 11 achieved SD, 6 achieved PR and 3 achieved CR. The efficacy evaluations for the cardiac metastases revealed that 3 patients achieved PD, 7 achieved SD, 4 achieved PR and 5 achieved CR. The efficacy evaluations for the other metastases revealed that 6 patients achieved PD, 6 achieved SD, 4 achieved PR and 3 achieved CR. The possible causes of death in the deceased patients were speculated. Based on the combination of their clinical manifestations and clinical treatment efficacy, 3 patients who had elevated N-terminal pro-B-type natriuretic peptide (NT-proBNP) levels but stable tumors were considered likely to have died of heart failure (Table 4).

**Table 4:** Evaluation of treatment efficacy in patients with cardiac metastases who continued treatment

ID	Age	Lymphoma	Status	Cardiac symptoms	Pericardial effusion	NT-proBNP	Clinical efficacy			Possible cause of death
							Original tumor	Cardiac metastases	Other metastases	
1	48	Yes	Died	Yes	No	142002.00	SD	SD	SD	Heart failure
2	51	Yes	Died	Yes	No	1934.00	PR	PR	SD	Tumor
3	60	No	Alive	No	No	-	CR	PR	CR	-
5	46	No	Died	Yes	No	857.70	PD	PD	PD	Tumor
6	21	No	Died	Yes	Yes	-	PR	PD	PD	Tumor
7	63	Yes	Died	No	No	-	CR	CR	CR	Tumor
8	73	Yes	Died	No	No	1767.00	SD	PR	SD	Tumor
9	66	No	Died	Yes	Yes	1305.00	\	\	\	Tumor
10	57	Yes	Alive	No	No	23.25	PR	CR	PR	-
11	67	Yes	Died	No	Yes	1426.00	PD	SD	PD	Tumor
12	66	Yes	Died	Yes	No	3211.00	\	\	\	-
13	29	No	Died	No	No	1918.00	SD	SD	PD	Tumor
14	35	No	Alive	Yes	Yes	294.30	SD	SD	SD	-
15	59	No	Died	No	No	-	SD	SD	PD	Tumor
18	69	No	Died	No	Yes	-	\	\	\	Tumor
19	52	No	Died	No	No	285.50	\	\	\	Tumor
23	60	No	Died	No	No	5234.00	SD	PD	PD	Heart failure
24	66	No	Alive	Yes	No	14393.00	SD	\	\	-
28	65	No	Died	No	No	-	SD	SD	SD	Tumor
29	72	No	Died	Yes	Yes	7808.00	SD	SD	SD	Heart failure
32	46	No	Alive	No	No	334.8	PR	PR	PR	-
33	19	No	Died	Yes	Yes	-	PD	\	\	Tumor
35	57	Yes	Alive	No	No	-	PR	CR	PR	-
36	13	Yes	Alive	No	No	-	CR	CR	CR	-
37	73	No	Died	Yes	No	-	SD	\	\	Tumor
40	66	No	Alive	No	Yes	-	SD	\	\	-
41	59	No	Alive	No	Yes	-	PR	CR	PR	-

Regarding cardiac metastases, five patients achieved CR after antitumor treatment (Table 5). The primary tumors in these patients included 4 lymphomas and 1 liver cancer. The patients with lymphoma all received chemotherapy. The patient with liver cancer underwent surgery to remove the tumor. The efficacy evaluations for the primary tumors revealed that 1 patient achieved CR and 4 achieved PR, including for the other metastases.

**Table 5:** Evaluation of treatment efficacy in 4 patients who achieved complete remission of their cardiac metastases

ID	Original tumor type	Cardiac metastasis site	Treatment type	Treatment plan	Clinical efficacy		
					Original tumor	Cardiac metastases	Other metastases
1	Lymphoma	Left ventricle	Chemotherapy	R-CHOP (8 cycles)	CR	CR	CR
2	Lymphoma	Pericardium	Chemotherapy	ABVD (6 cycles)	PR	CR	PR
3	Lymphoma	IVS & Pericardium	Chemotherapy	R-DA-EPOCH (8 cycles)	PR	CR	PR
4	Lymphoma	Pericardium	Chemotherapy	BFM-90/95 (stage I)	PR	CR	PR
5	Liver cancer	Right ventricular	surgery	\	SD	CR	PR

## Discussion

Cardiac tumors are rarely seen clinically and are more likely to be found postmortem, mainly because the symptoms of metastatic cardiac tumors are insidious and are not focused on in daily treatment. In this study, we collected, summarized and analyzed the clinical data of 41 patients with cardiac metastases to preliminarily describe the clinical features and treatment outcomes of these tumors. One-fourth of the patients had cardiac metastasis at the time when the primary tumor was diagnosed. More than half of the patients had cardiac metastasis within 6 months after the primary tumor diagnosis. Some of the patients with primary tumors evaluated as PD were found to have cardiac metastasis at the same time. When the primary tumor is evaluated as PD, an assessment of the heart may help to recognize cardiac metastasis in the early stage, although more samples are needed to confirm this recommendation.

The pathological types of cardiac metastases are diverse and depend on the nature of the primary tumor. In this study, one-fourth (10 of 41) of patients were observed to have lymphoma as the primary tumor. The second most common type of primary tumor was lung cancer (7 of 41), followed by esophageal cancer, cervical cancer, renal cancer, melanoma, colorectal cancer, osteosarcoma, soft tissue tumors and other unclassified primary tumors. As lymphoma is a non-solid tumor of lymph-hematopoietic origin, it has been reported that the heart seems to be more often involved in non‐Hodgkin's lymphomas, and the pericardium is more often infiltrated in Hodgkin's lymphoma [18, 19]. Thus, whether lymphomas are more likely than other tumor types to spread to the heart through the bloodstream should be of greater concern. For solid tumors, it has been reported that up to 10% of bronchogenic tumors have atrial invasion, and lung cancer is the most common solid primary tumor that spreads to the heart [7, 15, 20]. In this study, lung cancer still accounted for the most frequent histologic types of cardiac metastases that were observed, which was consistent with previous literature. The number of cardiac metastases and their sites were also recorded. The majority of them were solitary, located in the ventricles or invading the pericardium, which was similar to the current reports [7, 20]. However, there is no association between cardiac symptoms and the sites or number of cardiac metastases. Instead, all patients with pericardial effusion after cardiac metastases had cardiac symptoms. Furthermore, a history of cardiovascular disease and cardiovascular risk factors did not seem to be associated with the development of cardiac metastases.

Echocardiography is a common method for assessing cardiac function. Echocardiographic imaging may show a thickened myocardium, an abnormal myocardial structure and abnormal contractility after cardiac metastasis [19, 20]. Although cardiac function after cardiac metastasis has been reported in the literature [21], preserved systolic function was found in carcinoid metastasis patients. However, little is known about the changes in cardiac function before and after cardiac metastasis. In the present study, pre- and postmetastatic echocardiography data were collected in some patients. In these data, although there was one patient with an increased heart rate after cardiac metastasis, there was no marked difference in heart rate overall. The volume of the heart could not be compared effectively because it varies according to the site of the metastatic tumor. All patients who had echocardiography showed valve regurgitation. Valves are an unusual target for metastases due to the lack of vessels and constant cusp motion [22]. However, intracavitary masses can impede blood flow and cause valvular dysfunction [23], which may explain why patients with cardiac metastasis have valve regurgitation. Cardiac metastases occurred with different sizes in any part of the heart, and myocardial thickening and valvular regurgitation were also observed under echocardiography, but findings on hemodynamic changes were not apparent. The LVEF in cardiac metastasis patients was in the normal range, which was similar to the results of Pandya et al. [21]. The E/e’ ratio can reflect cardiac diastolic function, and a value above 12 is considered abnormal diastolic function [24, 25]. Compared with echocardiography data before cardiac metastases, three patients displayed E/e’ > 12 after cardiac metastasis but normal ejection fraction, which suggested the potential possibility of diastolic insufficiency. Altogether, the impact on cardiac function seems to be compensable in the early stage of cardiac metastasis.

Prolonging the survival of oncology patients has always been a common goal. The occurrence of cardiac metastasis indicated that the tumor was in the terminal stage. The 2022 ESC guidelines on cardio-oncology hold the view that systemic chemotherapy is needed for the treatment of cardiac metastases, but a lack of evidence to support its benefits and not much explanation is given in the guidelines [10]. To the knowledge, this is the first study to focus on the response of cardiac metastases and prognosis after continued antitumor treatment. In this study, survival analysis showed that patients who continued treatment after developing cardiac metastases had remarkably improved survival compared with those who withdrew from treatment, regardless of the treatment regimen. Notably, half of the patients received chemotherapy alone, but this study could not evaluate which type of treatment most effectively prolonged patient survival. Although there was no significant association between cardiac metastasis response and survival after treatment, the median survival time was longer in patients whose cardiac metastasis responded to treatment. Generally, it is beneficial for oncology patients to continue treatment after developing cardiac metastases.

Of the patients who had efficacy evaluations, when primary tumors appeared to recede, there were corresponding remissions or stabilization of the cardiac metastases and other metastases. When the primary tumors stabilize or progress, cardiac metastases and other metastases also appear to stabilize or progress. Thus, it was tentatively concluded that when cardiac metastases responded to a therapeutic regimen, their responses were accompanied by primary tumor responses. Among patients with cardiac metastases, there were 5 in whom the efficacy of treatment was evaluated as CR; 4 of these patients had lymphoma. The primary tumors were also evaluated as achieving CR or PR. For lymphoma patients, active chemotherapy after cardiac metastasis can still achieve good results. Therefore, after cardiac metastasis occurs, systemic therapy to address the primary tumors remains important, and a cardiac response can lead to favorable curative effects and prolong the survival period.

Theoretically, myocardial replacement with tumor cells may eventually cause heart failure [26]. The death of tumor patients after cardiac metastasis has also been speculated; there were 3 patients with high levels of NT-proBNP who presented chest tightness and shortness of breath and died of heart failure. There was a concern that some patients who showed symptoms of heart failure and elevated NT-proBNP but normal ejection fraction had the possibility of ejection fraction-preserved heart failure, but none of them received preventive heart failure treatment. According to the 2022 AHA Guidelines on Heart Failure [27], for patients with cancer-related cardiac risk, cardiac function assessment before therapy and cardiac function monitoring are highly recommended, but the benefits from pharmaceutical prevention with β-blockers and ACEIs/ARBs need more evidence. In cancer patients with cardiac metastases, cardiac function assessment and monitoring are needed for the early recognition of heart failure, along with low cardiotoxicity medications. Pharmaceutical prevention to reduce death from cardiovascular events still deserves more practice and investigation. Unfortunately, due to the concealed symptoms of cardiac metastasis and the incompleteness of existing NT-proBNP data, the risk of cancer patients dying from heart failure after cardiac metastasis needs further research.

## Conclusion

In conclusion, continuing antitumor therapy after the development of cardiac metastases can significantly prolong patient survival and can benefit patients. Cardiac metastases and primary tumors respond consistently to antitumor therapy. The risk of death due to heart failure in cancer patients with cardiac metastases needs to be further investigated.

### Study strengths and limitations

The strengths of this study include the first to show that continuing antitumor therapy after the development of cardiac metastases can benefit patients, and effective cardiac metastasis responses are accompanied by primary tumor responses. However, there are some limitations to the present study. First, cardiac metastatic cancer is rare, and the number of samples collected in this study was small, which was a major limitation; therefore, this study focused more on clinical characteristics and prognosis rather than on the analysis of risk factors. Our next study will include multicenter data to expand the sample size for more in-depth research. Second, this study initially found that continuing antitumor therapy after the development of cardiac metastasis could prolong the survival of patients, but we could not determine which specific regimen was superior due to the limited samples at present. Future studies will explore the impact of treatment option selection on the prognosis of patients with cardiac metastatic cancer based on expanded samples.

## Data Availability

The datasets used and/or analyzed during the current study are available from the corresponding author upon reasonable request.

## Abbreviations

HR: Heart rate
Lad: Left atrial diameter
LVd: Left ventricular diameter
IVSd: Interventricular septum diameter
PLVWd: Left ventricular posterior wall diameter
CO: Cardiac output
LVEF: Left ventricular ejection fraction
E/e’: Early diastolic transmitral flow velocity/early diastolic mitral annular velocity ratio
VR: Valvular regurgitation
